# Biochemical Approach to Poly(Lactide)–Copper Composite—Impact on Blood Coagulation Processes

**DOI:** 10.3390/ma17030608

**Published:** 2024-01-26

**Authors:** Zdzisława Mrozińska, Marcin H. Kudzin, Michał B. Ponczek, Anna Kaczmarek, Paulina Król, Agnieszka Lisiak-Kucińska, Renata Żyłła, Anetta Walawska

**Affiliations:** 1 Łukasiewicz Research Network—Lodz Institute of Technology, 19/27 Marii Sklodowskiej-Curie Str., 90-570 Lodz, Poland; zdzislawa.mrozinska@lit.lukasiewicz.gov.pl (Z.M.); anna.kaczmarek@lit.lukasiewicz.gov.pl (A.K.); paulina.krol@lit.lukasiewicz.gov.pl (P.K.); agnieszka.lisiak-kucinska@lit.lukasiewicz.gov.pl (A.L.-K.); renata.zylla@lit.lukasiewicz.gov.pl (R.Ż.); anetta.walawska@lit.lukasiewicz.gov.pl (A.W.); 2 Department of General Biochemistry, Faculty of Biology and Environmental Protection, University of Lodz, 90-236 Lodz, Poland; michal.ponczek@biol.uni.lodz.pl

**Keywords:** antimicrobial, blood coagulation, composite, copper, melt-blown, poly(lactide), magnetron sputtering

## Abstract

The paper presents the investigation of the biological properties of Poly(Lactide)—Copper composite material obtained by sputter deposition of copper onto Poly(lactide) melt-blown nonwoven fabrics. The functionalized composite material was subjected to microbial activity tests against colonies of Gram-positive (*Staphylococcus aureus*), Gram-negative (*Escherichia coli*, *Pseudomonas aeruginosa*) bacteria, *Chaetomium globosum* and *Candida albicans* fungal mold species and biochemical–hematological tests including the evaluation of the Activated Partial Thromboplastin Time, Prothrombin Time, Thrombin Time and electron microscopy fibrin network imaging. The substantial antimicrobial and antifungal activities of the Poly(Lactide)–Copper composite suggests potential applications as an antibacterial/antifungal material. The unmodified Poly(Lactide) fabric showed accelerated human blood plasma clotting in the intrinsic pathway, while copper plating abolished this effect. Unmodified PLA itself could be used for the preparation of wound dressing materials, accelerating coagulation in the case of hemorrhages, and its modifications with the use of various metals might be applied as new customized materials where blood coagulation process could be well controlled, yielding additional anti-pathogen effects.

## 1. Introduction

Nonwoven materials are gaining a lot of interest due to their unique characteristics, including light weight, large surface area, high porosity as well as air permeability and ease of processing [1,2], as well as due to the economic aspect and compliance with the assumptions of a circular economy. As a result, nonwovens have been increasingly used for a wide scope of applications, including various medical products, such as hygienic products, scaffolds for tissue engineering as well as wound dressings [3].

As far as wound dressings and other fibrous based medical devices (such as implants or scaffolds) are concerned, nonwovens ensure an open structure suitable for drainage of exudates and thus limit the risk of infection [4]. Moreover, owing to their three-dimensional, fibrous structure, nonwovens mimic the architecture of a natural tissue and provide a proper matrix for its regeneration [3,5]. High porosity of nonwovens enables the growth of a newly formed tissue within its structure and allows for the proper transport of nutrients and waste removal [5]. Therefore, nonwovens may be used for the regeneration of different types of tissues, for example skin and epithelial tissue [5,6,7,8,9,10,11,12,13,14,15].

Up to now, the majority of medical nonwovens have been produced by electrospinning [3,5]. However, this method is difficult to up-scale due to non-repeatable results, high production costs, low production capacity, practically no manufacturing capabilities. Therefore, melt blowing is gaining a lot of attention, since it offers an industrial scale production of fibrous materials at low cost, high throughput and with good reproducibility [12,16].

Medical nonwovens may be produced from different fibers, including natural and synthetic polymers. Currently a lot of attention is given to biodegradable polymers, among which polylactic acid (PLA) is one of the most common and extensively researched [16,17,18,19,20]. PLA degrades mainly through hydrolysis and the resulting degradation products may be eliminated by means of natural biological processes, without any adverse effects [16,18,19,20]. Moreover, PLA exhibits numerous advantages from the medical point of view, such as biocompatibility, biodegradability, bioreabsorbability, thermoplastic processability and eco-friendliness [16,18,19,20]. Therefore, PLA is widely used for medical applications, including wound management [19,20].

During the last few decades, the use of nonwoven wound dressings has been vastly studied and multiple different approaches have been proposed, including antibacterial and active substance (API)-releasing materials [3,21,22,23,24,25,26,27,28,29,30,31,32,33,34,35,36,37,38,39,40]. The incorporation of antimicrobial agents into wound dressings is vastly researched, since wounds are susceptible to microbial contamination, which may result in severe complications [3]. Hence, the prevention of secondary infection of wounds is of the utmost importance.

As the continuation of our research program directed at antibacterial composites [41,42,43,44], we present here the paper on PLA–Cu composites consisting of a hemostatic matrix [39,45,46,47,48,49] and hemostatic metal [50,51,52,53,54,55,56].

Between different antimicrobial agents, copper is gaining a growing attention [57,58,59,60,61,62,63,64]. The biocidal effect of copper may be associated with several different mechanisms [62,63,64]. These include the displacement of essential metals from their native binding sites [64]. Another explanation of copper’s toxicity towards microorganisms is the denaturation of nucleic acids or changes in their conformational structure [63,64]. These may be caused, for instance, by binding to or disordering of helical structures and by cross-linking between and within nucleic acid strands [63]. Furthermore, the antibacterial properties of copper may be associated with the alteration of proteins and inhibition of their biological assembly and activity [63,64]. Other proposed mechanisms involve the plasma membrane permeabilization, membrane lipid peroxidation, oxidative phosphorylation, osmotic balance or ligand interactions [63,64].

At the same time, copper exhibits lower toxicity in comparison to many other heavy metals, such as silver [61,65,66]. This is associated with the fact that, despite the high sensitivity of microorganisms towards copper, human tissue does not exhibit susceptibly to it [64,65,66]. Moreover, copper occurs naturally in plants, as well as in animal and human tissues [60]. It is an essential element, which has multiple important functions in terms of human health [60,62,65,67,68]. Therefore, it is considered that the deficiency of copper is more problematic than its toxicity [60]. This is related to the fact that the human body is able to metabolize copper [60]. Additionally, there is a variety of different mechanisms at the cellular, tissue and organ levels, which act as a protection against copper toxicity [60]. Furthermore, copper is considerably cheaper than silver, which is one of the most popular antimicrobial agents [62,67].

In terms of wound management, copper is believed to be a crucial element related to the wound-healing process [69,70]. According to the literature, copper enhances the angiogenesis process, as it induces the generation of the vascular endothelial growth factor (VEGF) [69,70]. Moreover, it was stated that copper increases the expression of integrin and positively affects the stabilization of fibrinogen and collagen [68,69,70]. Finally, copper plays an important role in matrix remodeling, cell proliferation and reepithelization due to the up-regulation of the activity of copper-dependent enzymes, proteins and polysaccharides [71,72,73]. The positive influence of copper on the process of wound healing has been confirmed in the literature [68,69,70,71,72,73,74].

In this study, the functionalization of melt-blown PLA fiber material by copper was performed using magnetron sputtering method. It is a versatile method enabling the deposition of well-controlled, thin films/layers on a modified substrate. Another advantage of magnetron sputtering is the fact that it is considered to be an eco-friendly, low-cost and waste-free technology [18,75,76]. Since both Cu and PLA derived materials can be used as worthy neutral or active components during the dressing and treatment of wounds [77], the authors decided to investigate the effect of PLA–Cu composites on the important process that initiates wound healing, i.e., blood coagulation. An important mechanism for the initiation of blood coagulation after an injury is contact with a negatively charged surface. This can be both wound contaminations as well as substances released from the body after an injury. The former may be sand or silica-rich earth, but also substances of pathogenic origin as a defensive evolutionary adaptation of terrestrial vertebrates to activate blood clotting, close a wound and stop blood loss. The latter are substances such as collagen and nucleic acids that come into contact with blood after the damage to the blood vessels. All of the above activate the blood contact factors of the intrinsic blood coagulation pathway present in the plasma by exposure to a negatively charged surface. These factors include factor XII (FXII), factor XI (FXI), plasma prekallikrein and high molecular weight kininogen (HK). Their collective activation, along with the entire intrinsic course of the coagulation cascade, can be monitored by activated partial thromboplastin time (aPTT) measurement [78]. The resulting fibrin network can be observed using scanning electron microscopy (SEM). Therefore, the authors decided to use these methods to further investigate such biochemical-hematological aspects of PLA–Cu composites.

## 2. Materials and Methods

### 2.1. Materials

#### 2.1.1. Polymers

- Poly(Lactic acid) (PLA) polymer was purchased from NatureWorks LLC (Minnetonka, MN, USA), type Ingeo™ Biopolymer 3251D, MFR = 30–40 g/10 min (190 °C/2.16 kg), T_mp_ = 160–170 °C in the form of granulate, and was used for the fabrication of nonwoven samples.

#### 2.1.2. Magnetron Usable Material

- Copper target was from Testbourne Ltd. (Basingstoke, UK) with 99.99% purity.

#### 2.1.3. Microbiological Strains

- The following bacterial and fungal strains were purchased from Microbiologics (St. Cloud, MN, USA):
  - *Escherichia coli* (ATCC 25922).
  - *Staphylococcus aureus* (ATCC 6538).
  - *Pseudomonas aeruginosa* (ATCC 27853).
  - *Chaetomium globosum* (ATCC 6205).
  - *Candida albicans* (ATCC 10231).

#### 2.1.4. Activated Partial Thromboplastin Time (aPTT), Prothrombin Time (PT) and Thrombin Time (TT)

Standard human blood plasma lyophilizates, aPTT reagent (Dia-PTT), PT reagent (Dia-PT) and TT regent (Dia-TT) and 0.025 M CaCl_2_ solution reagent were purchased from Dia-PTT (Diagon Kft, Budapest, Hungary) and a coagulometer (K-3002 OPTIC, KSELMED^®^, Grudziądz, Poland) was used for measurements. The reagents were prepared before the measurements according to the manufacturer’s instructions.

### 2.2. Methods

#### 2.2.1. PLA–Cu Composites Synthesis

##### PLA Nonwoven Fabrics

Poly(Lactic acid) nonwovens were fabricated by the melt-blown technique. A one-screw laboratory extruder (Axon, Limmared, Sweden), with a head with 30 holes of 0.25 mm diameter each, was used. Processing parameters for fabrication of Poly(Lactic acid) nonwoven samples are presented in Table 1.

##### Magnetron Sputtering Modification of Poly(Lactide) Nonwovens

The PLA nonwoven samples were modified using a direct current (DC) magnetron sputtering system produced by P.P.H. Jolex s. c. (Czestochowa, Poland). Magnetron DC sputtering parameters applied for the modification of nonwovens are presented in Table 2.

#### 2.2.2. PLA–Cu Composite Physical Characterization

Atomic Absorption Spectrometry with Flame Excitation (FAAS)

Determination of the copper content in composite samples was performed using a single-module Magnum II microwave mineralizer from Ertec (Wroclaw, Poland) and a Thermo Scientific Thermo Solar M6 (LabWrench, Midland, ON, Canada) atomic absorption spectrometer equipped with a 100 mm titanium burner, coded lamps with a single-element hollow cathode, background correction: D2 deuterium lamp.

The total copper content of the sample *M* (mg/kg; ppm) was calculated according to Equation (1) [79]:

(1) $$M=\frac{C_{i}\times V}{m_{i}}\;\left[\frac{\mathrm{mg}}{\mathrm{kg}}\right]$$

where:

*C_i_*—metal concentration in the tested solution (mg/L);

*m_i_*_—_mass of the mineralized sample (g);

*V*—volume of the sample solution (mL).

##### Microscopy Analysis

The morphology of the investigated samples was assessed using a VHX-7000N digital microscope (Keyence, Osaka, Japan). The applied magnification was equal to 500× and 2500×.

##### Specific Surface Area and Total Pore Volume Analysis

The specific surface area was determined by the Brunauer, Emmet and Teller method (BET). Measurements were carried out on an Autosorb-1 apparatus (Quantachrome Instruments, Boynton Beach, FL, USA) using nitrogen as a sorption agent and an adsorption isotherm at 77 °K. Prior to the analysis, the samples were dried at 105 °C for 24 h and degassed at room temperature. In each experiment, approximately 2 g of a given sample was weighed and used. Measurements were made in duplicate, and the results were presented as a mean value.

#### 2.2.3. PLA–Cu Composite Biological Characterization

##### Antimicrobial Properties

The antibacterial and antifungal activity of PLA–Cu^(t)^ fabrics were tested according to the EN ISO 20645:2006 [80] and EN 14119: 2005 [81] standards, respectively, against *E. coli* (ATCC 25922), *S. aureus* (ATCC 6538) and *Ch. globosum* fungus (ATCC 6205), analogously to our previous works [42,43]. The following concentrations of inoculum were used: *E. coli*—CFU/mL = 1.3 × 10^8^, *S. aureus*—CFU/mL = 1.9 × 10^8^, *Ch. globosum*—CFU/mL = 2.5 × 10^6^.

##### Biochemical–Hematological Properties

1. Activated Partial Thromboplastin Time (aPTT)

Standard human blood plasma lyophilizates were dissolved in 1 mL deionized water. A piece of the studied fabric (1 mg square slice) was added to each 200 μL plasma sample, vortexed and incubated for 15 min at 37 °C. For aPTT measurements, the Dia-PTT (kaolin and cephalin) reagent was resolved and 0.025 M CaCl_2_ solution reagent prepared, according to the manufacturer’s instruction. APTT measurements were performed on a coagulometer for each sample: 50 μL of plasma sample and 50 μL of suspension of Dia-PTT were introduced into a measuring cuvette and placed in the thermostat of the coagulometer at 37 °C. The mixture was left for 3 min, then the measurement was started by adding 50 μL of 0.025 M CaCl_2_ solution to the cuvette. Unused fabric remaining at the bottom of the incubation tubes along with the remaining plasma was primed for polymerization by adding the appropriate volume of reagents (Dia-PTT), incubated as previously described and made for polymerization by adding CaCl_2_ solution. Additional samples containing PLA material alone with plasma to which only CaCl_2_ solution was added for polymerization were also prepared. Similarly, the samples were prepared for the measurements of the remaining times.

2. Prothrombin Time (PT) and Thrombin Time (TT)

For PT, cuvettes with 100 μL of plasma were incubated at 37 °C in the thermostat of the coagulometer for 2 min (37 °C), after which 100 μL of Dia-PT was added and the measurement was started. Dia-PT contained tissue thromboplastin from rabbit brain, calcium ions and preservative, and was shaken each time before adding to obtain a homogeneous suspension. For TT, cuvettes were placed in the thermostat at 37 °C and 50 μL of plasma was introduced and incubated for 2 min. A total of 100 μL of Dia-TT was then added to the plasma and the time of fibrin clot formation was measured. All such samples were frozen (−80 °C) for storage and shipment for SEM.

The SEM (scanning electron microscope) analysis of Poly(Lactide) fiber materials and protein fibrin from associated blood plasma was performed on a scanning electron microscope model Quanta 200 (FEI company, Hillsboro, OR, USA) equipped with a Q150R S vacuum sputtering machine. The surface of each preparation was sprayed with a conductive substance (gold). The SEM microscopic analysis was carried out in a high vacuum using the energy of the probe beam 20 ekV. Magnification was 1000×.

## 3. Results and Discussion

### 3.1. Magnetron Sputtering Modification of Poly(Lactide) Nonwovens

**Figure 1:**
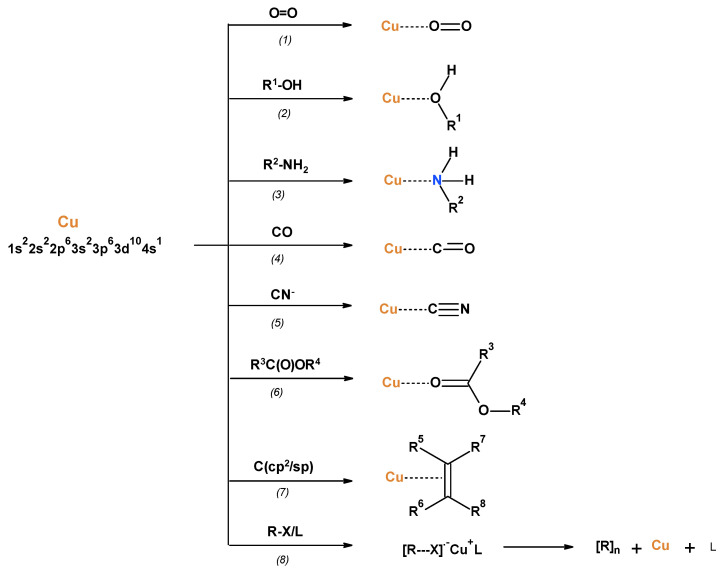
Metallic copper interaction/chemisorption with compounds: *(1)* oxygen [86]; *(2)* water and alcohols [87,88,89]; *(3)* ammonia [90,91,92] and hydrazine [93]; *(4)* carbon oxide [94,95,96]; *(5)* cyanides [95]; *(6)* carboxylic acids [93,97,98,99,100,101,102,103]; *(7)* sp/sp^2^ hydrocarbons [104,105]; *(8)* alkyl iodides [106] and in copper-mediated Living Radical Polymerizations [83,84,85].

The PLA samples were modified by surface deposition of metallic copper using a direct current (DC) magnetron sputtering system. Copper electron configuration 1s^2^ 2s^2^ 2p^6^ 3s^2^ 3p^6^ 3d^10^ 4s^1^ 4p^0^p^0^p^0^ enables its electro-donor, as well electro-acceptor, reactivity [82]. Therefore, metallic copper can chemisorb the array of small molecules and ions, and also serves as an electron donor in the copper-mediated Living Radical Polymerizations [83,84,85] (Figure 1). In these reactions, copper can serve as an electron acceptor or as an electron donor reagent.

**Figure 2:**
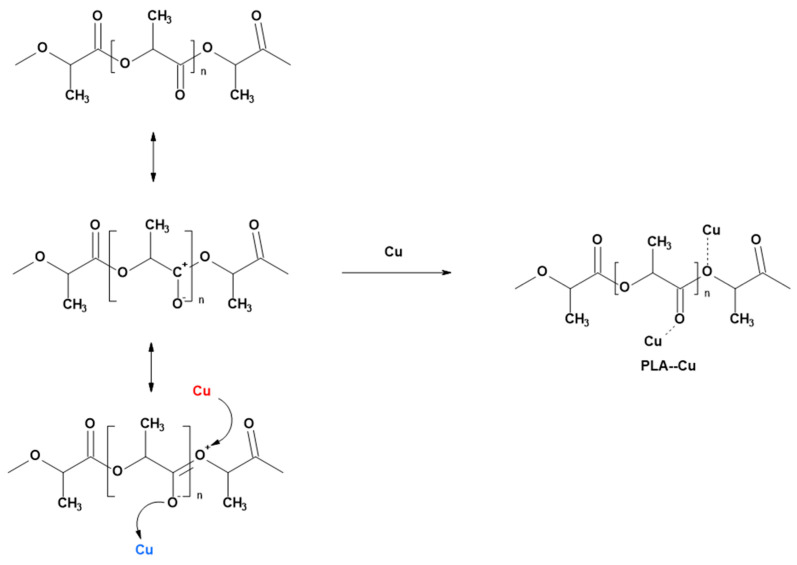
Putative mechanism of the formation of PLA–Cu interface (**Cu** acts as electron donor, **Cu**—acts as electron acceptor).

The copper properties summarized in Figure 1 suggest the formation of PLA–Cu composites with copper-polylactide bonds, which are illustrated schematically in Figure 2. Thus, some copper atoms deposited on the PLA surface (layers or islands) can interact with carbonyl oxygen acting as an electro-donor and forming a covalent Cu-O^−^ bond in the form of free-radical complex.

It seems natural that copper atoms, both chemisorbed or dispersed on the PLA surface, retain their reactivity (Figure 1) and therefore influence the biological properties of composites.

### 3.2. Physical Characteristics of PLA–Cu Composites

#### 3.2.1. Atomic Absorption Spectrometry with Flame Excitation (FAAS)

The copper contents in the examined samples were assessed by the FAAS method and are presented in Table 3. The results of the determination of copper content in the PLA–Cu^(t)^ composites show that the Cu concentration depends on the applied magnetron sputtering deposition time (PLA–Cu^(5)^: 5 min—0.85 g/kg (0.013 mol/kg); PLA–Cu^(10)^: 10 min—13.36 g/kg (0.21 mol/kg); PLA–Cu^(20)^: 20 min—32.92 g/kg (0.52 mol/kg)). The obtained results correspond to our previous studies [66]. The largest (in terms of quantity) increase in the amount of copper deposited on the substrate was observed while transcending 5 min. For a PLA–Cu^(5)^ sample, the copper content increased drastically by over 16 times.

#### 3.2.2. Microscopy Analysis

**Figure 3:**
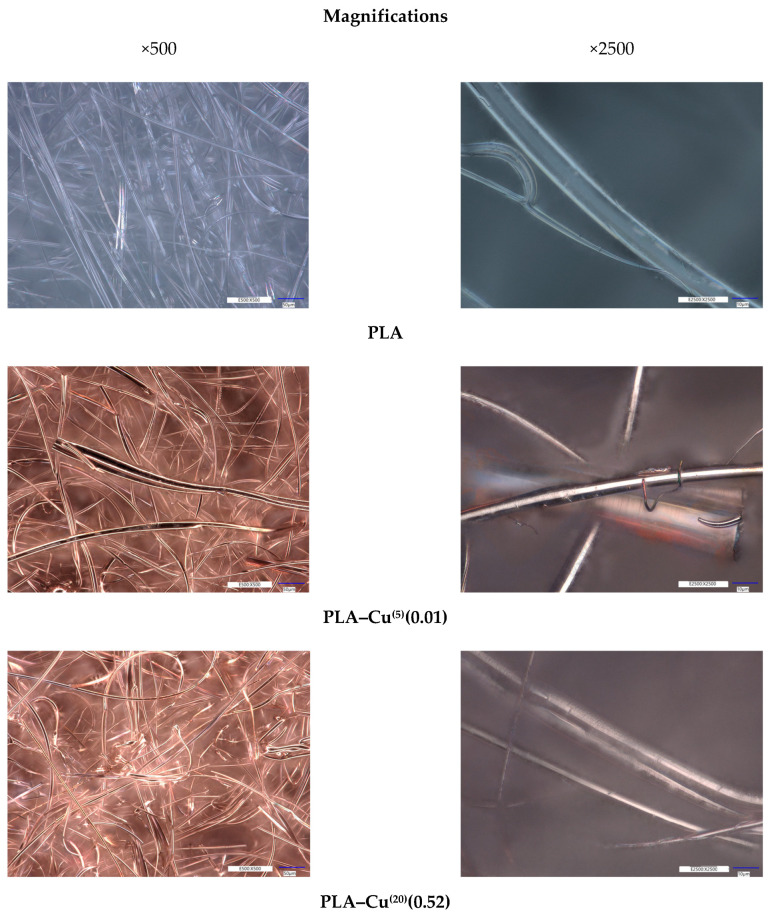
Optical microscopy images (magnifications: ×500, ×2500) of the surface structure of samples before and after the modification processes.

The microscopic images of the investigated samples obtained from the digital optical microscope are presented in Figure 3. It may be observed that the obtained copper coatings are uniform and cover not only the surface of the fibers, but the coating is also present in between the fibers and fills in the void spaces. However, the fibrous structure of the PLA nonwoven is preserved. The observations under the higher magnification showed that the PLA fibers were not damaged during the magnetron deposition process.

SEM micrographs of PLA and composites PLA–Cu^(10)^(0.16) and PLA–Cu^(30)^(0.43) were presented in earlier work [96].

#### 3.2.3. Specific Surface Area and Total Pore Volume Analysis

**Figure 4:**
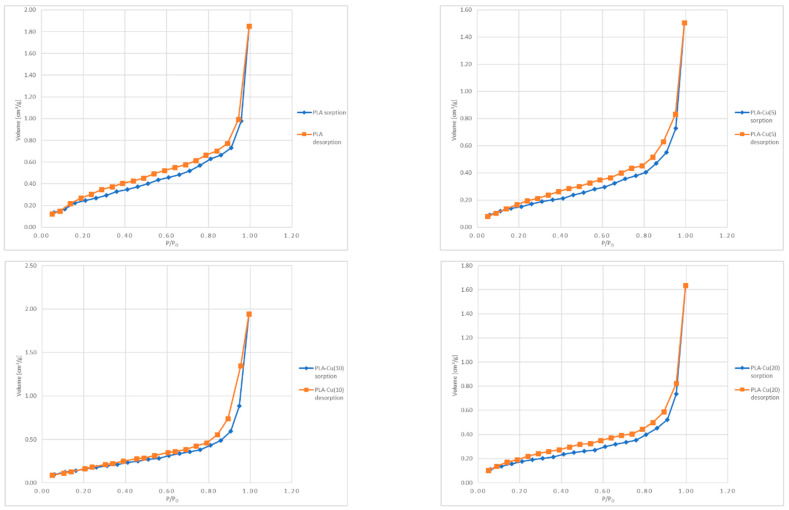
The N_2_ adsorption–desorption isotherms obtained for the investigated samples.

Figure 4 presents the N_2_ adsorption–desorption isotherms of the investigated samples. The shape of the obtained isotherms resembles the type II isotherm according to the IUPAC classifications of physisorption isotherms, i.e., the sigmoid or S shaped [107,108,109]. This type of isotherm is typical for monolayer–multilayer sorption on nonporous or macroporous surfaces [107,108]. Moreover, for all of the samples the appearance of a hysteresis loop was observed, that is usually associated with the presence of the mesoporosity and the capillary condensation in mesopores [107,108]. It may be noticed that the amount of adsorbate increased exponentially with growing pressure for all of the examined samples. For the low relative pressure, the increase in the amount of adsorbate was slow, while in the range close to p/p_0_ = 1 a rapid increase in the amount of adsorbed nitrogen occurred. This may be due to the fact that nitrogen molecules diffused firstly into the micropores at low pressure, and then were adsorbed in a monolayer and in subsequent layers at higher pressure [95]. However, it must be noted that the so-called “knee” or Point B, i.e., the beginning of the middle, almost linear, section [93], is not very distinctive. The more gradual curvature, especially in the case of the PLA–Cu^(10)^ sample, may be observed, that suggests the overlap of the monolayer formation and the beginning of the multilayer adsorption [107]. The most distinctive Point B was observed for the unmodified PLA. The shape of the hysteresis loops resembles the H3 type [107,108], which is typical for slit-shaped pores [109], and may occur when the macropores are not fully filled with the pore condensate [107].

The Total Pore Volume and specific surface area of the Poly(Lactide) melt-blown nonwoven sample (PLA) and Poly(Lactide)–Copper composite material are presented in Table 4.

The specific surface area of the Poly(Lactide) material sample (PLA) was equal to 0.9721 [m^2^/g]. The copper magnetron modification of Poly(Lactide) nonwoven resulted in a sudden decline to values 0.65–0.64 (m^2^/g) for its composites PLA–Cu^(5)^(0.01)–PLA–Cu^(20)^(0.52). The decrease of the observed specific surface area for the modified samples PLA > PLA–Cu^(n)^(mc) may be related to the lower mesoporosity of the composites. These were confirmed by the Total Pore Volume, which decreased from 3.858 × 10^−3^ (cm^3^/g) for the PLA nonwoven fabric to 2.01–2.24 × 10^−3^ for the PLA–Cu^(n)^(mc) sample. The rapid decrease of the SSA and TPV factors of the starting PLA during copper sputtering suggests a covering of the structural holes of the polymer by deposited copper atoms. Consequently, small changes of these factors during prolonged deposition of Cu can be related to the appearances of additional flat layers of copper coating on the PLA surface in the magnetron sputtering process. This is in agreement with the results obtained from the digital microscopy analysis, which revealed that the copper coating was deposited not only on the surface of the fibers, but also in between the fibers, filling the void spaces.

### 3.3. Biological Properties

The antibacterial activity of copper, resulting from its chemical properties (Figure 1), is well documented in the literature against numerous different strains and for all chemical states, i.e., elemental, oxides, ions as well as complexes [59,60,61,62]. Copper, as a redox active metal, exerts also a substantial role in the oxidative defense system [57,58].

#### 3.3.1. Antimicrobial Properties

The Poly(Lactide) nonwoven (PLA) and Poly(Lactide)–Copper composites (PLA–Cu^0(t)^) were subjected to antimicrobial activity tests against Gram negative (*E. coli*, *P. aeruginosa*), Gram positive (*S. aureus*) and a colony of *Ch. globosum* and *C. albicans* (Table 5 and Table 6). Results of these studies prove that the modification of nonwoven fabrics material with Cu provides antimicrobial properties against representative microorganisms (Table 5 and Table 6), expressed by inhibition zones of bacterial and fungal growth on Petri dishes. For a Poly(Lactide) nonwoven fabric sample without copper modification, a visible growth of microorganisms, covering the surface of the control sample (PLA), was observed. The results indicate that copper biocidal surfaces are effective against Gram-positive and Gram-negative bacteria and representative species of fungi. The mechanism responsible for the observed effect is the so-called “contact killing”. The microbes in contact with the copper present on the surface of the examined samples suffer rapid membrane damage and DNA degradation [110,111,112,113,114].

According to the literature, the “contact killing” occurs due to the cellular damage resulting from the direct physical interaction between microbes and a surface coated with metallic copper [62]. The mechanism of “contact killing” is associated with the release of copper ions from the surface and their accumulation in the membrane [115,116]. This results in the destabilization of the membrane due to the decrease in its potential and finally leads to the membrane damage [115,116]. As a consequence, copper ions enter and accumulate within the cell [102,103], which triggers the following effects. First of all, the reactive oxygen species in the form of hydroxyl radicals are generated due to the Fenton-like reactions [117,118,119]. The newly formed free radicals may then cause protein and lipid oxidation [104]. Moreover, they may damage the DNA structure and cause its degradation [115,116]. The released copper ion itself may interact with metal-binding sites of enzymes and cause their inhibition [117,118,119]. For example, copper ions damage Fe–S clusters in cytoplasmic hydratases by coupling to sulfur followed by the displacement of iron [117,118,119]. This results in the depletion of sulfhydryl groups such as cysteine of glutathione [117,118,119]. Similarly, copper ions may compete with other metal ions for divalent cation-binding sites on proteins [119]. Finally, the copper ions may influence the microbial respiration, which may be suppressed due to the cytochrome inhibition [115,116].

The obtained results correspond to our previous studies [41,42,43]. Antimicrobial tests revealed the dependence of the amount deposited copper on the substrate in between on the antibacterial properties. Samples modified with time over 5 min using DC magnetron sputtering exhibit an antimicrobial character, due to the higher amount of copper detected on their surface, than the samples exposed for less than 5 min.

#### 3.3.2. Biochemical–Hematological Properties

##### Blood Plasma Clotting—Activated Partial Thromboplastin Time (aPTT)

**Figure 5:**
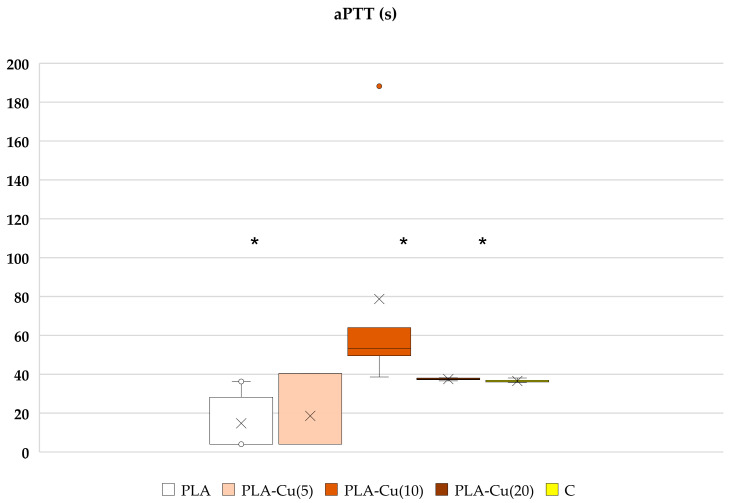
Effect of selected materials on aPTT. The samples: PLA, PLA–Cu^(5)^(0.01); PLA–Cu^(10)^(0.21); PLA–Cu^(20)^(0.52) and C—plasma control (*n* = 6, 5, 5, 6, 6, respectively; data differ significantly from that which is normally distributed in a Shapiro–Wilk test, * *p* < 0.05 in one-tailed Mann–Whitney U Test relative to C). Results are presented as mean (×), median (horizontal line), range (bars) and interquartile range (box), the orange dot is outlier maximum extreme value.

**Figure 6:**
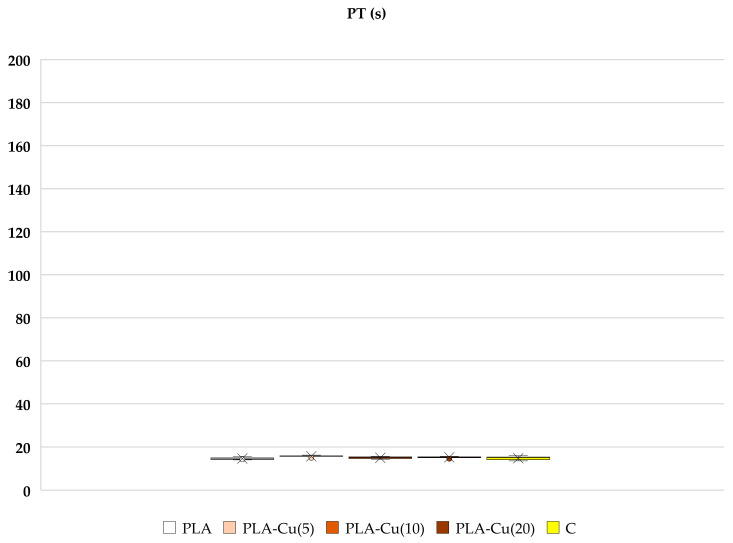
Effect of selected materials on PT. The samples: PLA, PLA–Cu^(5)^(0.01); PLA–Cu^(10)^(0.21); PLA–Cu^(20)^(0.52) and C—plasma control (*n* = 6, 5, 5, 6, 6, respectively; data differ significantly from that which is normally distributed in a Shapiro–Wilk test). Results are presented as mean (×), median (horizontal line), range (bars) and interquartile range (box).

**Figure 7:**
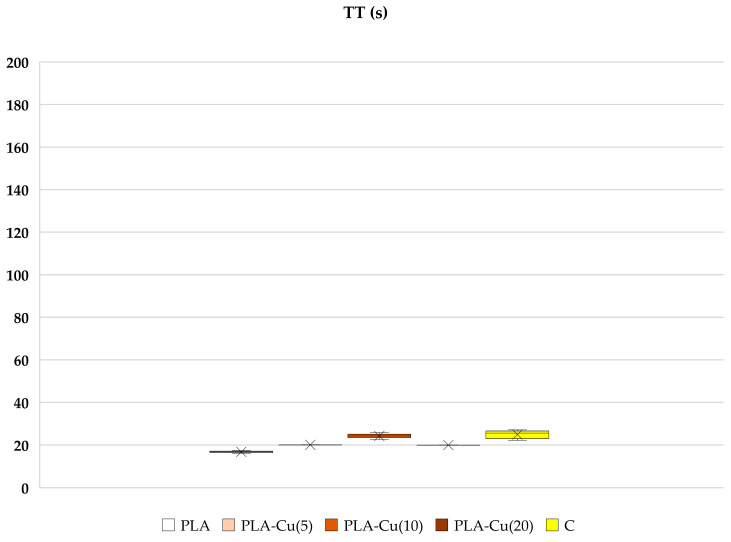
Effect of selected materials on TT. The samples: PLA, PLA–Cu^(5)^(0.01); PLA–Cu^(10)^(0.21); PLA–Cu^(20)^(0.52) and C—plasma control (*n* = 6, 5, 5, 6, 6, respectively; data differ significantly from that which is normally distributed in a Shapiro–Wilk test). Results are presented as mean (×), median (horizontal line), range (bars) and interquartile range (box).

Poly(Lactide) nonwoven fabrics without magnetron sputtering modification (PLA) exhibit a shorter clotting time initiated by contact (intrinsic pathway) measured as the activated partial thromboplastin time (aPTT), whereas the modification of nonwoven fabrics with Cu provides a disappearance or even a reversal of such an effect. PLA–Cu^(5)^(0.01) still shows some shortening of the clotting time, but this is statistically not significant; PLA–Cu^(10)^(0.21) causes a statistically significant prolongation of aPTT, while the clotting time for PLA–Cu^(20)^(0.52) differs only a little from the results obtained for plasma alone (Figure 5). The materials do not significantly affect the prothrombin (PT) and thrombin times (TT), see Figure 6 and Figure 7.

Only PLA–Cu^(10)^(0.21) led to some prolongation of contact clotting activation in human blood plasma, as indicated by the longer aPTT. There was no such effect on PT, so only contact factors (XI, XII, HK) could probably be adsorbed on the Cu surface and their diminishing concentration in plasma thus was responsible for aPTT prolongation. No change of the coagulation cascade in extrinsic pathway (PT) was observed, therefore, the elements of extrinsic and both pathways were not affected. Such a conclusion could also be supported by the few studies describing the influence of transition metals on contact factors, including examining copper, nickel, cobalt and zinc, which showed the influence on aPTT and the binding of factors XI, XII and HK in human plasma [105,106]. Regardless of this observation, other PLA modifications with copper had no negative influence on coagulation, so they could potentially be used as dressing materials, taking into account their additional properties examined in the current study.

The aPTT is an indicator of the efficiency of the intrinsic (contact) mechanism of generating blood plasma coagulation through the formation of thrombin and the generation of a fibrin clot. The aPTT determines the time of formation of a fibrin clot after maximum activation on a negatively charged surface: in the presence of kaolin (silicates and cephalin) contact factors—FXII, FXI, PK and HK initiate the blood coagulation cascade. The tested plasma is incubated with a suspension of kaolin and cephalin—the activators of the intrinsic coagulation pathway by contact with a negatively charged surface, and then after the addition of Ca^2+^ ions, the time of clot formation is measured. FXII and HK play an important role in blood clotting activation on the negatively charged surface, where FXII is autoactivated in the contact complex with the participation of the previously mentioned components. PLA in its coiled polymer structure contains many oxygen groups of a carbonyl group on which, due to the electronegativity difference within the carbon atom, polarization occurs, and a dipole is formed with a partial negative charge on the oxygen atom. Many such atoms in close proximity to each other are good activators of the intrinsic pathway of blood coagulation, as in the case of silica, polyphosphates, nucleic acids and other similar substances. To date, PLA has not been well studied in this regard. Our results showed that it is an activator of blood coagulation as it contributes to the shortening of the clotting time in the aPTT test, which measures the path of intrinsic blood coagulation activation. Cu reverts this effect by contact factor absorption, however, with the appropriate copper content, it does not cause negative adverse effects in the form of negative inhibition and prolongation of clotting.

##### Prothrombin Time (PT) and Thrombin Time (TT)

No statistical effect was observed when PT and TT times were measured, where blood clotting is triggered by extrinsic pathway (tissue factor—thromboplastin, calcium ions), or thrombin, respectively (Figure 6 and Figure 7). Extrinsic pathway starts with tissue factor, which activates factor VII leading to fibrin formation, when prothrombinase complex is formed. Thrombin time uses thrombin as the final activator of fibrinogen to fibrin conversion. Results suggest that fabrics have no impact on factors participating in the above mentioned activation, indicating here additionally the importance of contact factors in the intrinsic way and the probable activation of blood coagulation on the negatively charged surface of PLA.

**Figure 8:**
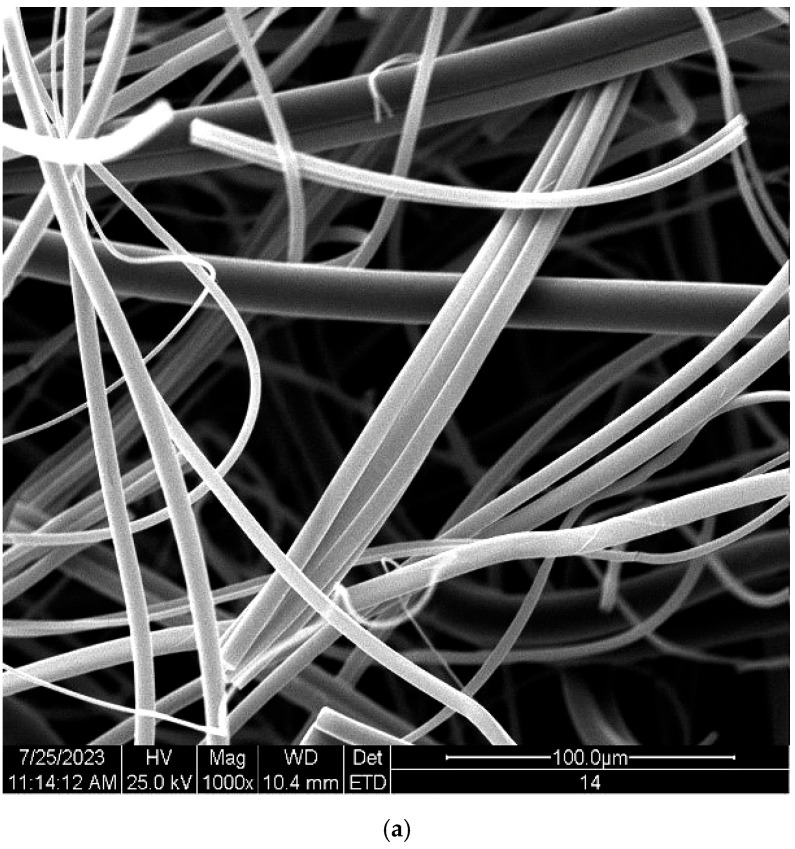

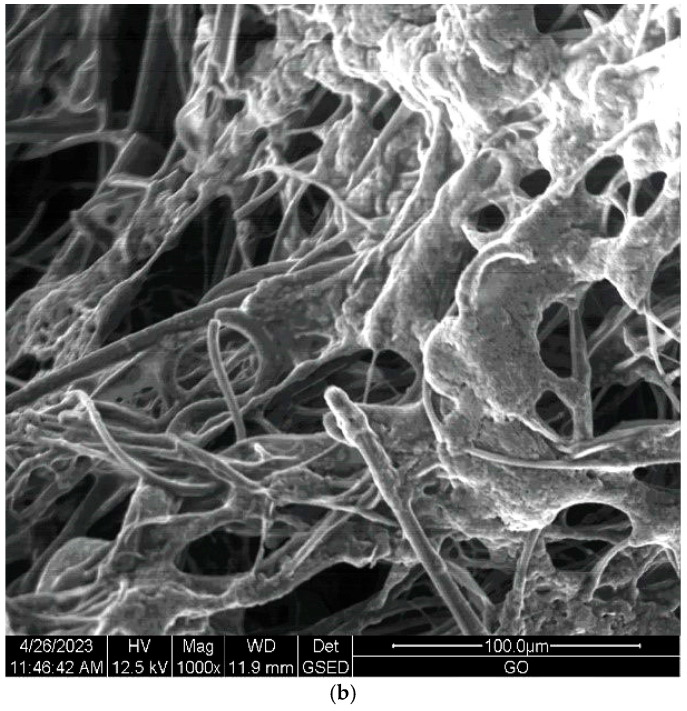
Scanning electron microscopy micrographs: (**a**) unmodified PLA nonwoven; (**b**) surface of PLA sample with associated fibrillar protein network, magnification: 1000×.

The observed shortening of the clotting time for PLA and its non-toxicity and biodegradability, on the one hand, would make it a good dressing material, accelerating blood clotting in the case of injuries and hemorrhages, but on the other hand, it poses a risk of unwanted clots when this material is used as a product for the preparation of endovascular implants or other medical inserts intended to cause unwanted thrombosis. Therefore, the modification by shielding of PLA material by various transition metal atoms, including copper, may be important in obtaining its novel derivatives with various new properties, including those that do not lead to the unwanted activation of blood coagulation. In the course of the current research, we have shown that coating PLA with a layer of copper eliminates the properties that activate blood coagulation, leads to a return to normal control values and may even slightly extend them, acting as an anticoagulant, which depends on the thickness of the metallic layer. For a sample with a thick Cu layer—PLA–Cu^(20)^(0.52), the activation effect disappears completely with a slight increase in clotting time (Figure 7). Additionally, changes in the network of forming fibrin can be observed, as has been demonstrated in SEM studies as the agglomerates of fibrin proteins on Poly(Lactide) fiber material (PLA) (Figure 8b).

## 4. Conclusions

The paper presents biological characterization of Poly(Lactide)–Copper composite materials obtained by sputter deposition of copper on the Poly(Lactide) melt-blown nonwoven fabrics. The functionalized composite materials were subjected to microbial activity tests against colonies of Gram-positive (*S. aureus*), Gram-negative (*E. coli*, *P. aeruginosa*) bacteria, fungal molds (*Ch. Globosum*, *C. albicans*) and biochemical–hematological tests including the evaluation of the activated partial thromboplastin time, prothrombin time, thrombin time and electron microscopy fibrin network imaging (SEM). The unmodified Poly(Lactide) fabric showed accelerated aPTT human blood plasma clotting in the intrinsic pathway, while copper plating abolished this effect by probable surface absorption of contact factors. No effect, regardless of modification, was observed in the case of the extrinsic pathway—PT and activation by thrombin—TT. Unmodified PLA itself could be used for the preparation of wound dressing materials, accelerating coagulation in the case of hemorrhages, and its modifications with the use of various transition metals might be a way to search for new materials where blood coagulation process could be well controlled, yielding additional anti-pathogen effects. The substantial antimicrobial and antifungal activities of PLA–Cu^0^ suggest potential applications as an antibacterial/antifungal material.

## Figures and Tables

**Table 1:** Melt-blown technique processing parameters applied for preparation of PLA nonwovens.

Melt-Blown Processing Parameters	
Temperature of the extruder in zone 1	195 °C
Temperature of the extruder in zone 2	245 °C
Temperature of the extruder in zone 3	260 °C
Head temperature	260 °C
Air heater temperature	260 °C
Air flow rate	7–8 m^3^/h
Mass per unit area of nonwovens	200 g/m^2 (a)^
Polymer yields	6 g/min

^(a)^ In the work [42]: 102 g/m^2.^.

**Table 2:** DC magnetron sputtering processing parameters applied for the modification of PLA nonwovens.

DC Magnetron Sputtering System Processing Parameters	
Target	Copper (99.99%)
Power discharge	0.5 kW ^(a)^
Power density	0.70 W/cm^2 (b)^
Working pressure	2.0 × 10^−3^ mbar
Working atmosphere	Argon
Distance between sample and target	15 cm
Deposition time	5 min; 10 min; 20 min
Sputtered sample size	60 cm × 20 cm

In the work [42]: ^(a)^ Power discharge 0.7 kW; ^(b)^ Power density 0.72 W/cm^2^.

**Table 3:** Results of the determination of copper content in tested samples.

Sample Name		S. d. Time	Cu Concentration				**Final Sample Name** PLA–Cu^(t)^(mc)
			This Work		Lit. Data [96]		
		[min.]	[g/kg]	[mol/kg]	[g/kg]	[mol/kg]	
PLA		-	-	-	0.004	0.00006	
PLA–Cu^(t)^	PLA–Cu^(5)^	5	0.85	0.013			PLA–Cu^(5)^(0.01)
	PLA–Cu^(10)^	10	13.36	0.21			PLA–Cu^(10)^(0.21)
	PLA–Cu^(10)^				9.91	0.16	PLA–Cu^(10)^(0.16)
	PLA–Cu^(20)^	20	32.92	0.52			PLA–Cu^(20)^(0.52)
	PLA–Cu^(30)^				27.89	0.43	PLA–Cu^(30)^(0.43)

S. d. time—sputtering deposition time t. Cu^(t)^(mc): t = 5, 10 and/or 20 min of copper sputtering, respectively; mc—molal concentration of copper on composite surface: 0.014, 0.21 or 0.52 (mol/kg), respectively. The results have been measured in triplicate and are presented as a mean value with ±deviation equal to approximately 2%.

**Table 4:** The specific surface area and total pore volume for unmodified PLA sample and copper composites PLA–Cu^(t)^.

Sample Name		Copper Concentration.	Specific Surface Area (SSA)	Total Pore Volume (TPV)
		Mol/kg	m^2^/g	cm^3^/g
PLA		-	0.9721	3.858 × 10^−3^
PLA–Cu^(n)^(mc)	PLA–Cu^(5)^(0.01)	0.01	0.6465	2.235 × 10^−3^
	PLA–Cu^(10)^(0.21)	0.21	0.6489	2.221 × 10^−3^
	PLA–Cu^(20)^(0.52)	0.52	0.6372	2.006 × 10^−3^

The results have been measured in duplicate and are presented as a mean value with ±deviation equal to approximately 2%.

**Table 5:** Results of antimicrobial activity tests of polylactide nonwoven PLA and Polylactide–Copper composites PLA–Cu^(t)^ on the basis of standard EN-ISO 20645:2006 [80] compared with the literature data [42].

Sample Name		Average Inhibition Zone (mm)				
		*E. coli*		*S. aureus*		*P. aeruginosa*
		This Work	Lit. [42]	This Work	Lit. [96]	
PLA		0		0		0
PLA–Cu^(n)^(mc)	PLA–Cu^(5)^(0.01)	0		0		0
	PLA–Cu^(10)^(0.16)		2		1	
	PLA–Cu^(10)^(0.21)	1		1		1
	PLA–Cu^(20)^(0.52)	2		1		1
	PLA–Cu^(30)^(0.43)		2		1	

The following concentrations of inoculum were used: *E. coli*—CFU/mL = 1.3 × 10^8^ (1.2 × 10^8^ in [93]); *S. aureus*—CFU/mL = 1.9 × 10^8^ (1.7 × 10^8^ in [96]).

**Table 6:** Results of antimicrobial activity tests of alginate composites on the basis of EN 14119: 2005 standard [81].

Sample Name		Average Inhibition Zone (mm)	
		*Ch. globosum*	*C. albicans*
PLA		0	0
PLA–Cu^(n)^(mc)	PLA–Cu^(5)^(0.01)	0	0
	PLA–Cu^(10)^(0.21)	no grow	no grow
	PLA–Cu^(20)^(0.52)	no grow	no grow

Concentration of inoculum [CFU/mL]: *Ch. globosum*—2.5 × 10^6^, *C. albicans*—0.8 × 10^8^.
